# Agonistic CD40 antibody induces immune-mediated liver damage and modulates tumor-induced myeloid suppressive cells

**DOI:** 10.1186/2051-1426-2-S3-P174

**Published:** 2014-11-06

**Authors:** Jose Medina-Echeverz, Chi Ma, Austin Duffy, David E Kleiner, Tim F Greten

**Affiliations:** 1 National Cancer Institute, USA; 2 NCI/NIH, USA

## 

Immune stimulatory monoclonal antibodies are currently evaluated as anti/cancer agents in clinical trials. While overall toxicity seems to be moderate, liver toxicities have been reported and are not completely understood. Based on the observation that CD11b^+^Gr-1^+ ^MDSC accumulate in the liver of tumor-bearing mice, we decided to study the effect of anti-CD40 treatment on hepatic MDSC and the liver immune microenvironment.

100 mg agonistic CD40 antibody (FGK-45) or isotype control IgG was injected into naïve and tumor-bearing mice. Liver enzymes, histologies and immune infiltrates were analyzed 24 hours after antibody administration. The effect of CD40 agonist was evaluated using bone marrow chimeras and genetically modified mouse strains *CD40^-/- ^*and *gp91^-/-^*. Mouse and human tumor-induced myeloid suppressive cells were cultured with CD40 agonists to address changes in their immune function.

Agonistic CD40 antibody caused liver damage into tumor-bearing mice in two unrelated tumor models and mice strains. Using bone marrow chimeras we could demonstrate that anti-CD40 induced hepatitis in tumor-bearing mice was dependent on the presence of CD40^+ ^immune cells. Anti-CD40 ligation-dependent liver damage was induced by the generation of reactive oxygen species. Furthermore, agonistic CD40 antibody resulted in increased CD80 and CD40 positive liver CD11b^+ ^Gr-1^+ ^immature myeloid cells. CD40 ligation on tumor-induced hepatic myeloid cells or CD14^+ ^HLA-DR^low/neg ^PBMC from cancer patients reduced their immune suppressor function.

Collectively, agonistic CD40 antibody activates the tumor-induced liver myeloid suppressive compartment, enhancing acute inflammation and thus liver damage. Our data in murine and human myeloid suppressive cells point towards a pivotal role for CD40 as a key molecule in modulating myeloid suppressive cell plasticity.

